# Shared decision making for anticoagulation reduces anxiety and improves adherence in patients with atrial fibrillation

**DOI:** 10.1186/s12911-023-02260-x

**Published:** 2023-08-22

**Authors:** Hsiao-Hui Chiu, Shih-Lin Chang, Hao-Min Cheng, Tze-Fan Chao, Yenn-Jiang Lin, Li-Wei Lo, Yu-Feng Hu, Fa-Po Chung, Jo-Nan Liao, Ta-Chuan Tuan, Chin-Yu Lin, Ting-Yung Chang, Ling Kuo, Chih-Min Liu, Yung-Nan Tsai, Yu-Ting Huang, Yuh-Lih Chang, Ju-Chieh Wung, Shih-Ann Chen

**Affiliations:** 1 Department of Nursing, Taipei Veterans General Hospital, Taipei, Taiwan; 2 Department of Nursing, National Taipei University of Nursing and Health Sciences, Taipei, Taiwan; 3 Division of Cardiology, Department of Medicine, Taipei Veterans General Hospital, No. 201, Sec. 2, Shih-Pai Road, Taipei, Taiwan; 4 School of Medicine, National Yang Ming Chiao Tung University, Taipei, Taiwan; 5 Heart Rhythm Center and Division of Cardiology, Department of Medicine, Taipei Veterans General Hospital, Taipei, Taiwan; 6 Department of Pharmacy, Taipei Veterans General Hospital, Taipei, Taiwan; 7 Department of Pharmacy, National Yang Ming Chiao Tung University, Taipei, Taiwan; 8 Institute of Pharmacology, College of Medicine, National Yang Ming Chiao Tung University, Taipei, Taiwan; 9 Cardiovascular Center, Taichung Veterans General Hospital, Taichung, Taiwan; 10 College of Medicine, Chung Hsing University, Taichung, Taiwan

**Keywords:** Oral anticoagulants, Atrial fibrillation, Anxiety, Shared decision making, CHADS-VASc scores, Stroke

## Abstract

**Background:**

Treatment with oral anticoagulants (OACs) could prevent stroke in atrial fibrillation (AF), but side effects developed due to OACs may cause patients anxiety during decision making. This study aimed to investigate whether shared decision making (SDM) reduces anxiety and improves adherence to stroke prevention measures in patients with AF.

**Methods:**

A one-group pretest–posttest design using a questionnaire survey was applied at the outpatient cardiology clinic between July 2019 until September 2020. A Patient Decision Aid (PDA) tool was used for the completion of the questionnaire survey after health education and counseling. Ten questions were included for patients’ recognition of SDM, and a 5-point scoring method was used, where “very much” was scored as 5 points, and “totally not” was scored as 1 point.

**Results:**

Fifty-two patients with AF were enrolled. In terms of patients’ recognition of SDM, points of more than 4.17 out of 5 were noted, indicating recognition above the level of “very much.” The patients’ anxiety scores before SDM were 3.56 (1.2), with a decrease of 0.64 points (*p* < 0.001) to 2.92 (1.3) after SDM. After SDM, the number of patients who decided to take OAC increased from 76.9% to 88.5%, and the 15.4% answering “unclear” decreased to 1.9% (*p* = 0.006). The patients’ anxiety levels after SDM were associated with gender (*p* = 0.025).

**Conclusions:**

The approach using SDM enhanced our understanding of the pros and cons of OAC treatment and, in patients with AF, decreased anxiety about therapeutic decisions and increased willingness to accept treatment options.

**Supplementary Information:**

The online version contains supplementary material available at 10.1186/s12911-023-02260-x.

## Introduction

In Taiwan, the incidence rates of atrial fibrillation (AF) are 1.68 per 1000 person-years for men and 0.76 per 100 person-years for women. The overall prevalence of AF is 1.4% in men and 0.7% in women [1]. Moreover, 16.5% of ischemic stroke cases are associated with AF, and the incidence of AF in people aged > 75 years accounts for more than 10% [2, 3]. With regard to the CHADS-VASc scores for stroke risk stratification, the assessment scores range from 0 to 9, with higher scores indicating greater risks. Patients scored as ≥ 1point are recommended to undergo treatment using oral anticoagulants (OACs) to prevent stroke episodes [4]. Nevertheless, patients still have anxiety and concerns about the potential side effects of anticoagulants. Indeed, a study by Polikandrioti showed that severe anxiety was reported by 34.9% of patients with AF, with higher levels in women (*p* = 0.022) and in elderly (*p* = 0.022) patients. Moreover, patients with an educational level of university or primary education had higher anxiety than those with a high school educational level (*p* = 0.025) [5].

Shared decision making (SDM) involves evidence-based medicine, in which physicians collaboratively help patients by integrating their medical specialty with the patient’s preference for collective discussion and selection of therapeutic options [6]. Owing to the considerations in OAC treatment, the guidelines suggest a class I recommendation for patients with AF and physicians to use shared decision-making for the prevention of stroke [4]. Research from Hendriks found that knowledge associated with the ability of anticoagulants to reduce stroke risk among patients with AF mostly came from information provided by medical personnel in shared medical decision making [7]. The aim of this study was to test whether SDM reduces anxiety and improves adherence to stroke prevention measures in patients with AF.

## Methods

### Study design and setting

The study applied the one-group pretest–posttest design using a questionnaire survey.

Data were collected from patients who visited the outpatient cardiology clinic at a Veteran General Hospital in northern Taiwan. Patients were diagnosed with AF and were recommended to take oral anticoagulants by the attending physician. Taipei Veterans General Hospital is a large integrated healthcare delivery system that provides comprehensive medical services to a population of more than 3 million people in Taiwan.

### Participants

Fifty-two patients with AF aged over 30 years, who visited the outpatient cardiology clinic between July 1, 2019 and September 30, 2020, were enrolled. The inclusion criteria were patients diagnosed with AF, who were recommended by the physician to take oral anticoagulants, and who agreed to receive health education guidance and complete a questionnaire survey after verbal explanation by the physician. The exclusion criteria included patients who did not need to take oral anticoagulants, those who were unable to communicate in Mandarin or Taiwanese, and those who did not intend to receive health education guidance or complete the questionnaire survey.

### Validity and reliability

1. PDA tool: The program titled “Shall I take oral anticoagulants to prevent stroke if I have AF?” has been created based on an evidence-based approach adopting the Taiwan Ministry of Health and Welfare’s Promotion Program for Shared Decision Making-Scholars and Experts in Cardiology (Additional file 1) [2, 3, 8–11]. The content includes: Introduction, Applicable subjects/applicable conditions, Briefing on illness or health issues, Briefing on medical options, Your preferable option at present, Comparison on options, Items concerned in options, as well as Intentness, Information awareness, Confirming medical measures, and More information. Cronbach’s Alpha was 0.90.
2. Consistency among research staff: The staff in charge of the study comprised three nurses from the outpatient cardiology clinic. With guidance and supervision from the attending physicians of the cardiology division, a consensus conference was conducted to confirm the implementation procedure and methods to practice the instructions for using auxiliary tools and to achieve consistent expression among the three staff.

### Recruitment

An advertisement for recruitment was put up in the waiting area of the outpatient cardiology clinic, with an incentivized invitation by the attending physicians to complete a questionnaire survey.

### Data collection

Following an explanation of the study objective and procedure of enrollment by the cardiology physicians, the patients provided verbal agreement, and were transferred to a quiet private space for health education. The research staff provided the decision-making auxiliary tool “Shall I take oral anticoagulants to prevent stroke if I have AF?” to conduct one-on-one professional counseling for approximately 20 to 30 min prior to completing the questionnaire survey.

### Statistical analysis

The data were input in Excel with an anonymized encoding process. The statistical software SPSS 20.0 (IBM SPSS Inc. Chicago Illinois) was used to perform statistical analyses. Statistically significant *p*-values were considered with a threshold for statistical magnitude, percentage, mean score, and standard deviation in the paired t-test and one-way analysis of variance.

## Results

### Participant characteristics

Fifty-two patients were enrolled in the study and had the following characteristics: The subjects for SDM were mostly the patients themselves (86.5%); most of the subjects were male (61.5%); most were > 65 years (57.5%); and the majority of the subjects had educational levels of college and above (51.9%) (Table 1). Moreover, stroke evaluation for patients based on CHADS-VASc score showed the order as 2 points (23.1%), 3 points (19.2%), and 4 points (13.5%), among which, “hypertension,”accounting for 57.7%,was in the majority, “female”accounting for 38.5%, followed by two items including “age ≧ 75 years,” and “with vascular disease,”, all accounting for 23.1%.

**Table 1:** Demographic characteristics of the participants

	n	(%)
Identity		
Self	45	(86.5)
Relative	7	(13.5)
Sex		
Male	32	(61.5)
Female	20	(38.5)
Age		
< 64 years	22	(43.5)
> 65 years	30	(57.5)
Education		
Less than Junior high school	16	(30.7)
Senior high school	9	(17.3)
More than College	27	(51.9)
CHADS-VASc score		
0 points	4	(7.7)
1 points	6	(11.5)
2 points	12	(23.1)
3 points	12	(19.2)
4 points	7	(13.5)
5 points	4	(7.7)
6 points	5	(9.6)
7 points	4	(7.7)
Congestive heart failure	11	(21.2)
Hypertension	30	(57.7)
Age≧ 75 years	12	(23.1)
Diabetes mellitus	8	(15.4)
Stroke/transient ischemic attack	4	(7.7)
Vascular disease	12	(23.1)
Age 65–74 years	15	(28.8)
Sex category (female gender)	20	(38.5)

### Patients’ recognition items for SDM

Ten questions were included for patients’ recognition items of SDM; a 5-point scoring method was used for “very much” scored as 5 points, “relatively much” as 4 points, “some” as 3 points, “a little” as 2 points, and “totally not” as 1 point. The recognition for the 10 questions was > 4.17 points, indicating that the recognition was greater than “very much.” The top three scores for recognition were in sequence of “Did SDM help you to consider the most important pros and cons? (4.35 points),” “Did SDM help you to consider the pros and cons? (4.33 points),” and “Did SDM help you to schedule the follow-up with the doctor? (4.31 points),” indicating a relatively higher recognition in helping the patients to understand the pros and cons of treatment. The lowest score included “Did SDM help you to realize that you have to make a decision? (4.17 points),” and “Did SDM help you to share your main concern with the doctor? (4.17 points),” indicating a patient’s lower recognition of confirmed decision-making after SDM (Table 2).

**Table 2:** Patient recognition items for SDM

Subject	M (SD)
Did SDM help you to realize that you have to make a decision?	4.17 (0.92)
Did SDM help you to make a better decision?	4.25 (0.84)
Did SDM help you to consider the pros and cons?	4.33 (0.79)
Did SDM help you to consider the most important pros and cons?	4.35 (0.76)
Did SDM help you to know that the decision depends on something that matters to you?	4.27 (0.87)
Did SDM help you to organize your thoughts during decision-making?	4.25 (0.81)
Did SDM help you to know the involvement during decision-making?	4.27 (0.74)
Did SDM help you to realize the questions that you wanted to ask the doctor?	4.27 (0.80)
Did SDM help you to share you main concerns with the doctor?	4.17 (0.96)
Did SDM help you to schedule the follow-up with the doctor?	4.31 (0.76)

*SDM* Shared decision making

### Patients’ anxiety about selection in medical decision making after SDM

A 5-point scoring method was used for anxiety, with “very much” scored as 5 points, “relatively much” as 4 points, “some” as 3 points, “a little” as 2 points, and “totally not” as 1 point. The patients’ anxiety score before SDM was 3.56 (1.2) points, and the anxiety score after SDM was 2.92 (1.3) points, showing a significant decrease of 0.64 points (*p* < 0.001, 95% confidence interval [CI]:0.338–0.932). This indicates that SDM reduces patient anxiety when receiving anticoagulant treatment.

### The influence of SDM on patients’ decision making

**Fig. 1:**
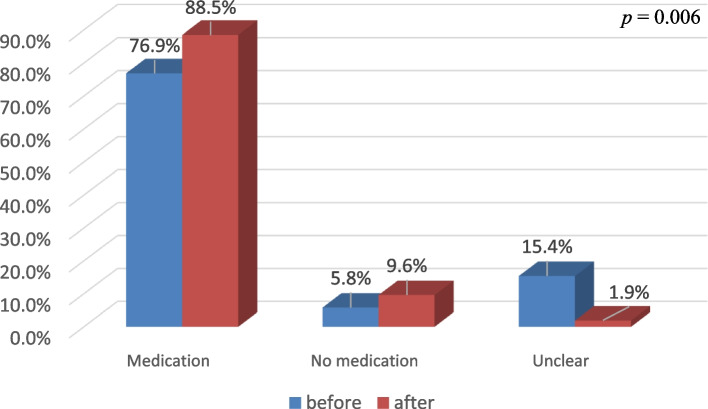
Impact of the SDM program on patients’ decision-making. Comparison of the difference in patients’ decision-making in treatment selection before and after SDM

Comparing the difference in patients’ decision-making in treatment selection before and after SDM, those who decided to take OACs (medication) increased from 76.9% to 88.5%, who decided not to take OACs (medication) increased from 5.8% to 9.6%, and decreased from 15.4% to 1.9% in those who were undecided (unclear) (*p* = 0.006) (Fig. 1).

### Factors associated with patients’ anxiety

Gender, education, and age were used as parameters to evaluate the factors associated with anxiety. Gender was associated with patients’ anxiety after SDM (*p* = 0.025) (Table 3).

**Table 3:** Factors associated with patient’s anxiety

Factors	Before SDM		After SDM	
	F	*P*	F	*P*
Sex	0.736	0.536	3.087	0.025^*^
Education	0.521	0.521	0.578	0.680
Age	0.167	0.670	1.256	0.301

One-way analysis of variance

*SDM* Shared decision making

^*^p-value < 0.05

## Discussion

### Participant characteristics

In this study, males accounted for 61.5%, and age ≥ 65 years accounted for 57.5% of the cases, which is consistent with the characteristics of the enrollment hospital as a veteran hospital. Moreover, the majority of participants had an educational level of college and above (51.9%), which is probably associated with the nature of the enrollment hospital, which is located in an urban area with higher educational levels.

### Patients’ recognition items of SDM

Patients’ recognition of the SDM provided by the medical team was scored as > 4.17 out of 5 points, indicating that the recognition was greater than “very much;” the top three scores in recognition showed that SDM helped patients to understand the pros and cons of treatment. Stacey et al. found an increased acknowledgment level in patients who used SDM compared to those who received routine nursing care (95%CI: 11.17–15.51), with lower uncertainty (95%CI: − 9.73 to − 4.78). Research from Hendriks found that the majority of the patients’ knowledge on the benefit of anticoagulants in reducing stroke risks in AF came from information provided by medical personnel in shared medical decision making [7]. Joseph-Williams et al. suggested that factors affecting the success of medical decision making involve consideration of the previous background knowledge of the case, the severity of the impact on quality of life of the case from medical options, as well as the stability of the patient’s illness [12]. The cases enrolled in this study were outpatients, and the majority were scored as 2 points (23.8%) in CHADS-VASc, indicating alower severity of illness with relatively stable conditions. Moreover, the majority of patients had an educational level of college and above (51.9%). Stacey found that SDM could increase the patients’ understanding of decision making, and minimize the conflict in decision making associated with ignorance, obscureness, and personal values. This enhanced participation in decision-making provides the patients with more accurate expectations by reinforcing the patient’s knowledge [13, 14]. In agreement with previous studies, SDM can help patients to understand the pros and cons of treatment.

### Influence of SDM on patients’ decision making

Cheng et al. found that anxiety decreased from 3.5 points to 2.1 points (*p* < 0.05) in patients with AF using SDM [15], which is consistent with the present study showing that SDM can reduce patients’ anxiety. Our study showed that the willingness to use medication increased from 76.9% to 88.5% in AF patients with SDM. Similarly, Shen et al. showed that the willingness to receive treatment for vertebral fracture increased from 68.1% to 97.9%, with an increase of 29.8%, indicating that the use of SDM was beneficial in enhancing the willingness to choose to undergo treatment. A recent study also demonstrated that SDM enhanced the quality of treatment and clinician satisfaction with no encounter duration [16, 17].

### Factors associated with patients’ anxiety

Among AF patients, increased anxiety was found in female, elderly, and patients with an educational level of university or primary education [5]. Anxiety after SDM was also found to be associated with gender in our study. During clinical counseling, females were found to actively ask more questions and require more time for instructing and explaining.

## Conclusion

SDM can increase the understanding of the pros and cons of OAC treatment, reduce anxiety, and increase selection willingness in decision making, and it induces clinically realistic benefits in patients with AF.

### Supplementary Information

**Additional file 1: Table 1.** The program of “Shall I take oral anticoagulants to prevent stroke if I have AF?” for shared decision making (SDM).

## Data Availability

All data generated or analysed during this study are included in this published article.

## Abbreviations

OACs: Oral anticoagulants
AF: Atrial fibrillation
SDM: Shared decision making
PDA: Patient Decision Aid
