# Supplementary material for: Shared decision making for anticoagulation reduces anxiety and improves adherence in patients with atrial fibrillation
Source: BMC Med Inform Decis Mak. 2023 Aug 22;23:163. doi: 10.1186/s12911-023-02260-x (PMC10463811; doi:10.1186/s12911-023-02260-x)
Supplement: Supplementary file 1 — Additional file 1: Table 1. The program of “Shall I take oral anticoagulants to prevent stroke if I have AF?” for shared decision making (SDM). [file 12911_2023_2260_MOESM1_ESM.docx]

Table 1. The program of “Shall I take oral anticoagulants to prevent stroke if I have AF?” for shared decision making (SDM).

**Shall I Take Oral Anticoagulants to Prevent Stroke if I Have AF**

**[Introduction]**

When you are confirmed by the physician’s diagnosis to have one type of arrhythmia – “atrial fibrillation”, such an arrhythmia can significantly increase the risk of stroke in the future, regardless of occasional or continued episodes. Taking anticoagulants may effectively reduce the risk for stroke episodes but can simultaneously increase the possibility for bleeding. The risk for stroke episodes varies from person to person, stroke risk can be obtained by calculation from the following table. After discussion with medical personnel, you may decide if you intend to use the medication according to your personal risk, conditions and opinion. During the process, please consult medical personnel if you have any enquiry.

**[Applicable cases/Applicable conditions]**

Patients with atrial fibrillation diagnosed by the physician, which may need to take anticoagulants to prevent stroke.

Your stroke risk score is points. (To be completed by medical personnel)

| **Risk Factor** | **Check the box if yes** | **Score** |
| --- | --- | --- |
| **With heart failure** | □ | 1 |
| **With hypertension** | □ | 1 |
| **Age ≥ 75 years old** | □ | 2 |
| **With diabetes** | □ | 1 |
| **With episodes of stroke/transient ischemic attack/thromboembolism** | □ | 2 |
| **With vascular diseases** | □ | 1 |
| **Age of 65-74 years old** | □ | 1 |
| **Female** | □ | 1 |
| **Total score** |  |  |

Patient number in stroke episodes among those who are with atrial fibrillation in various stroke risk scores per 1,000 population per year in Taiwan[1]

**
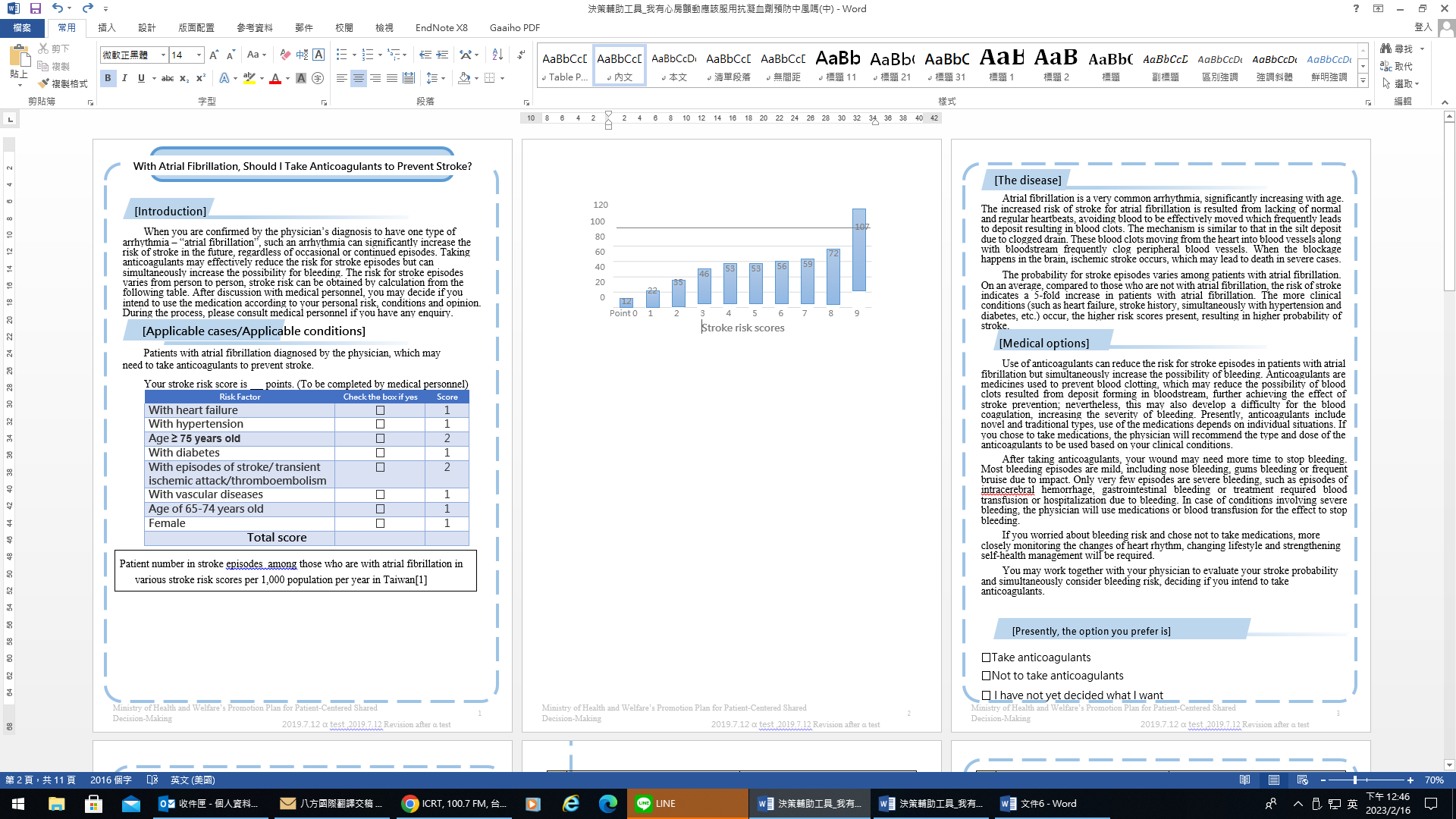
**

**[The disease]**

Atrial fibrillation is a very common arrhythmia, significantly increasing with age. The increased risk of stroke for atrial fibrillation is resulted from lacking of normal and regular heartbeats, avoiding blood to be effectively moved which frequently leads to deposit resulting in blood clots. The mechanism is similar to that in the silt deposit due to clogged drain. These blood clots moving from the heart into blood vessels along with bloodstream frequently clog peripheral blood vessels. When the blockage happens in the brain, ischemic stroke occurs, which may lead to death in severe cases.

The probability for stroke episodes varies among patients with atrial fibrillation. On an average, compared to those who are not with atrial fibrillation, the risk of stroke indicates a 5-fold increase in patients with atrial fibrillation. The more clinical conditions (such as heart failure, stroke history, simultaneously with hypertension and diabetes, etc.) occur, the higher risk scores present, resulting in higher probability of stroke.

**[Medical options]**

Use of anticoagulants can reduce the risk for stroke episodes in patients with atrial fibrillation but simultaneously increase the possibility of bleeding. Anticoagulants are medicines used to prevent blood clotting, which may reduce the possibility of blood clots resulted from deposit forming in bloodstream, further achieving the effect of stroke prevention; nevertheless, this may also develop a difficulty for the blood coagulation, increasing the severity of bleeding. Presently, anticoagulants include novel and traditional types, use of the medications depends on individual situations. If you chose to take medications, the physician will recommend the type and dose of the anticoagulants to be used based on your clinical conditions.

After taking anticoagulants, your wound may need more time to stop bleeding. Most bleeding episodes are mild, including nose bleeding, gums bleeding or frequent bruise due to impact. Only very few episodes are severe bleeding, such as episodes of intracerebral hemorrhage, gastrointestinal bleeding or treatment required blood transfusion or hospitalization due to bleeding. In case of conditions involving severe bleeding, the physician will use medications or blood transfusion for the effect to stop bleeding.

If you worried about bleeding risk and chose not to take medications, more closely monitoring the changes of heart rhythm, changing lifestyle and strengthening self-health management will be required.

You may work together with your physician to evaluate your stroke probability and simultaneously consider bleeding risk, deciding if you intend to take anticoagulants.

**[Presently, the option you prefer is]**

□Take anticoagulants

□Not to take anticoagulants

□I have not yet decided what I want

**Now, please use the following four steps to help you make a decision**

**Step one-Option comparison**

|  | **Take anticoagulants** | **Not to take anticoagulants** |
| --- | --- | --- |
| **Must**  **Do** | - The must-do regardless of which kind of anticoagulant you take:  1. Required to continuously take medications, voluntary drug withdrawal may influence the effect.   Required to voluntarily inform the physician of presently taking anticoagulants in seeking medical attention.   1. Reduce fall episodes or any activities prone to be hurt.  - The additional must-do when taking traditional anticoagulants:  1. Regularly receive blood draw for tests, following up drug efficacy and side effects. 2. Preferably avoid foods that may affect the efficacy of such a traditional anticoagulant. *   ※Even taking medications, you are still required to regularly perform measurement for blood pressure and blood sugar, quit smoking, pay attention to early signs of stroke, revisit for follow-up, as well as live a regular and healthy lifestyle. | 1. Strengthenself-health management, including: regularly perform measurement for blood pressure and blood sugar, quit smoking, do exercises; revisit for follow-up, monitor heart rhythm. 2. More alert to early signs of stroke episodes, normally are sudden symptoms including:    1. Weakness or numbness on one side of arm, leg or the face    2. Slurred speech, or insufficient tongue mobility    3. Temporary loss of or blurred vision with one eye or both eyes    4. Vertigo, unsteady gait    5. Unconsciousness, etc.   Seek medical attention earliest possible for the above conditions, even though such symptoms may recover soon. |
| **Preventive**  **Effect[1,2]** | Research in Taiwan indicates, compared to traditional anticoagulants, use of novel anticoagulants provides better effect in stroke prevention.  There are roughly 23-30 patients experienced stroke per 1,000 population per year among those who used novel anticoagulants; there are roughly 35 patients experienced stroke per 1,000 population per year among those who used traditional anticoagulants.  Nevertheless, different novel anticoagulants almost have the same efficacy in stroke prevention. | 1. Stroke probability rises with the increased risk scores. Research in Taiwan indicates, based on 2-3 points, stroke episodes occur averagely among 35-46 people per 1,000 population per year. 2. Blood clots blockage in the brain or other organs results in stroke, complications or death. |
| **Bleeding**  **Risk[1,2]** | Bleeding episodes required blood transfusion or hospitalization vary among different types of anticoagulants.  There are roughly 15-21 patients experienced severe bleeding per 1,000 population per year among those who used novel anticoagulants; there are roughly 33 patients experienced severe bleeding per 1,000 population per year among those who used traditional anticoagulants.  Severe cerebral hemorrhage is very uncommon, there are roughly 7 patients per 1,000 population per year among those who used novel anticoagulants; there are roughly 14 patients per 1,000 population per year among those who used traditional anticoagulants. | Without the risk of bleeding as taking medications may have. |

* Foods with high vitamin K (leafy green vegetables such as spinach, broccoli, lettuce, as well as egg yolk, liver, etc.) may affect the efficacy of some anticoagulants. If you eat such foods, preferably have consistent daily amount. Do not abruptly eat too much or abruptly avoid eating. [^3^] Consult your medical personnel in case of relevant questions.

**Step two-What are the items you care about in choosing medical options? What is the degree you care about?**

Example description for circling:

Please refer to the following items, circling the situation closest to yours. If you care about “may reduce stroke possibility” more than “worry about bleeding problem caused by medications”, please circle the number according to the degree you care about in the side-column for “Compared to bleeding, reduced risk of stroke is more important for me.”, a greater number represents the higher degree you care about.

| Reasons to accept taking anticoagulants | Closer Same Closer | | | | | | | Reasons not to take anticoagulants |
| --- | --- | --- | --- | --- | --- | --- | --- | --- |
| 2. Compared to bleeding, I care more about stroke. | 3 | 2 | 1 | 0 | 1 | 2 | 3 | Compared to stroke, I care more about cerebral hemorrhage or gastrointestinal bleeding caused by the medications. |

| Reasons to accept  taking anticoagulants | Closer Same Closer | | | | | | | Reasons not to take  anticoagulants |
| --- | --- | --- | --- | --- | --- | --- | --- | --- |
| 1. I care much about stroke. | 3 | 2 | 1 | 0 | 1 | 2 | 3 | With the lower stroke risk score, my possibility for stroke is not high. |
| 1. Compared to bleeding, I care more about stroke. | 3 | 2 | 1 | 0 | 1 | 2 | 3 | Compared to stroke, I care more about cerebral hemorrhage or gastrointestinal bleeding caused by the medications. |
| 3.I can regularly revisit, taking medications on schedule. | 3 | 2 | 1 | 0 | 1 | 2 | 3 | I worry much about being unable to take medications on schedule. |
| 4. Paying attention todietary restrictions will not cause any inconvenience to my daily life. | 3 | 2 | 1 | 0 | 1 | 2 | 3 | It is difficult to comply with or remember dietary restrictions, being troublesome. |
| 5.Most of my regular activities are not too intense or angerous. | 3 | 2 | 1 | 0 | 1 | 2 | 3 | Most of my regular activities are more intense or highly dangerous. |
| 6.Other considerations: |  |  |  |  |  |  |  | Other considerations: |

**Step three-Have you already understood the information provided above?**

1. I am with atrial fibrillation, compared to other people with normal heart rhythm, I have a higher probability for stroke?

□True□False□Uncertain

1. Use of anticoagulants can reduce the risk of stroke for patients with atrial fibrillation?□True□False □Uncertain
2. Use of anticoagulants may increase the risk for bleeding?

□True□False □Uncertain

1. I am with atrial fibrillation, both novel and traditional anticoagulants can reduce the probability for stroke episodes?

□True□False □Uncertain

If any answer of the above items is “False” or “Uncertain”, please consult your medical personnel for further explanation.

**Step four-Did you confirm your medical option now?**

1.I have clearly known the options for stroke prevention □Yes □No

2.I have clearly known the benefits and drawbacks for these options.

□Yes □No

3.For the problems presently encountered, I have received sufficient knowledge and recommendation. □Yes □No

4.I have confirmed the option I want. □Yes □No

5.I：□intend to take anticoagulants□do not want to take anticoagulants

6.I am unable to make a decision at present.

□I would try to discuss again with others (such as family members, friends, etc.).

□I would like to make a further discussion with my physician before making a decision.

7.I have other questions to be discussed with my physician (such as: subsequent issues, other queries):

**[Knowing about further information and resources]**

If you like to know about further information, please refer to the following websites:

- Taiwan Heart Rhythm Society’s Health Education Information Webpage for Atrial Fibrillation<http://www.afhealthcare.org.tw/>
- Taiwan Heart Foundation<http://www.tsoc-thf.org.tw>

**[Reference]**

1. Chao TF, Lip GY, Liu CJ, Tuan TC, Chen SJ, Wang KL, Lin YJ, Chang SL, Lo LW, Hu YF, Chen TJ, Chiang CE, Chen SA. Validation of a Modified CHA2DS2-VASc Score for Stroke Risk Stratification in Asian Patients With Atrial Fibrillation: A Nationwide Cohort Study. Stroke.2016;47(10):2462-2469
2. Chan YH, See LC, Tu HT, et al. Efficacy and Safety of Apixaban, Dabigatran, Rivaroxaban, and Warfarinin Asians With Non valvular Atrial Fibrillation. J Am Heart Assoc. 2018;7(8):e008150.
3. Drug-nutrition interactions: Coumadin and vitamin K. Warren Grant Magnuson Clinical Center, National Institutes of Health.
4. 2014 AHA/ACC/HRS Guideline for the Management of Patients With Atrial Fibrillation: Executive Summary. Journal of the American College of Cardiology,64(21).
5. 2016 ESC Guidelines for the management of atrial fibrillation developed in collaboration with EACTS. European Heart Journal. 2016;37:2893–2962.
6. 2016 Guidelines of the Taiwan Heart Rhythm Society and the Taiwan Society of Cardiology for the management of atrial fibrillation. Journal of the Formosan Medical Association. 2016;115:893-952.
7. Ruff CT, Giugliano RP, Braunwald E, Hoffman EB, Deenadayalu N, Ezekowitz MD, Camm AJ, Weitz JI, Lewis BS, Parkhomenko A, Yamashita T, Antman EM. Comparison of the efficacy and safety of new oral anticoagulants with warfarin in patients with atrial fibrillation: a meta-analysis of randomised trials. Lancet. 2014;383(9921):955-62.
